# Designing interview guides on stress and coping related to parenting pre‐teen children: an example from a hermeneutic phenomenological study

**DOI:** 10.1002/nop2.778

**Published:** 2021-07-12

**Authors:** Sarah Oerther

**Affiliations:** ^1^ Saint Louis University School of Nursing St. Louis MO USA

**Keywords:** child behaviour, hermeneutics, instrument development, nurse–patient interactions, parenting, phenomenology, philosophy, qualitative approaches, research methods

## Abstract

**Aims:**

To develop a semi‐structured interview guide on stress and coping related to parenting pre‐teen children for a hermeneutic phenomenological research study.

**Design:**

Hermeneutic phenomenological research approach which describes the development of an interview guide with semi‐structured questions.

**Methods:**

Ovid MEDLINE, CIHAHL, ERIC, SCOPUS, Web of Science, JSTOR, Education Source, PsyINFO and ProQuest were searched to identify possible interview guides with questions related to stress and coping. The literature was searched in 2019 and included manuscripts from 1970–2019. An initial interview guide was constructed. Mock interviews were used to confirm the rigour of the guide.

**Results:**

The final outcome was a semi‐structured interview guide on stress and coping related to parenting pre‐teen children.

**Conclusion:**

The development of this semi‐structured interview guide is relevant to hermeneutic phenomenological researchers who are interested in discovering how personal background meanings and interpersonal concerns shape parents’ day‐to‐day stress appraisals and coping with parenting pre‐teen children.

**Impact**

This semi‐structured interviewing guide can be an impactful tool for researchers to use to understand how personal background meanings and interpersonal concerns shape background meanings and concerns related to stress and coping with parenting pre‐teen children.

## 1 INTRODUCTION

Hermeneutic phenomenological research is an established qualitative approach that is well matched to reveal self‐world relations and varied patterns and transitions in human meanings and practices (Benner & Wrubel, 1989; SmithBattle, 2018). Hermeneutic phenomenological research is contextual and offers a way “to study persons, events, and practices in their own terms” (Benner, 1994, p. 99). A major premise of the approach is that humans primarily relate to the world by engaging in practical activities; that is, people learn how to act and relate to others by absorbing the meanings embedded in everyday activities (Benner & Wrubel, 1989; SmithBattle, 2018). Data for employing hermeneutic phenomenological research are generally collected using semi‐structured interviews with the aid of interview guides.

With semi‐structured interviews, researchers develop an a priori set of questions as an interview guide before conducting the interview, and then during the semi‐structured interview the set of questions guide, but do not dictate, the interview (SmithBattle, 2014). Every semi‐structured interview guide must be based on the study aims and the sampling criteria established for the study. Emerging scholars may find it difficult to develop interview guides for hermeneutic phenomenological research. This paper demonstrates the construction of a stress and coping interview guide for parenting pre‐teen children. In addition, suggestions to enhance the rigour of interview guides are also discussed.

## 2 BACKGROUND

### 2.1 Rene Descartes

Rene Descartes (1596—1,650), a French philosopher, believed that the universe operated similar to a machine, and if the laws of the universe could be understood, then actions of the human body could be deduced and repaired, like a machine (Berman, 1981). His philosophical reasoning resulted in the view that the body and mind are separate (Magee, 1987). Descartes was the first to depict the body as a machine, which contributed to the mechanistic view of the body (Leder, 1992). The living patient's body is divided into its component parts and their interactions, and viewed in a machine‐like fashion (Leder, 1984). This contributed to the notion in philosophy that the body and mind, the subject and the object, and the person and the world are separate and distinct entities, which came be to known as Cartesian dualism (Guignon, 1983).

Knowledge began to be conceptualized by some medical professionals as something that is entirely in the mind and separate from the body (Leder, 1984). A patient was viewed as subject, and the world, or the environment, as objective. A consequence of this reasoning was that healthcare professionals became fixated with the idea of a person as a collection of variables, such as “anxiety” or “self‐esteem,” which measured as context‐free traits to be joined according to theories that can be discovered through the scientific method (Leonard, 1989).

### 2.2 Martin Heidegger

In contrast to Descartes, Martin Heidegger, a German philosopher writing during the twentieth century, proposed that knowledge was not limited to conscious thought (McConnell‐Henry et al., 2009; Moran & Mooney, 2002). Rather, Heidegger claimed that Descartes’ focus on universal objective knowledge overlooked practical or pre‐theoretical understanding that is not held in the mind but is embedded in everyday practices (Guignon, 1983). For instance, when people drive a car, they are often unaware of all the embodied skills they use to drive the car (Benner & Wrubel, 1989). A skilled driver barely notices actions such as turning the steering wheel, staying in the lane or hitting the brake, even though the driver might do this almost fifty times on a trip to the store. These actions are automatic and escape the driver's conscious thought. Instead, the skilled driver's attention is usually focused on manoeuvring around other cars to get to the destination, or the driver is focused on the conversation with a passenger in the car (Benner & Wrubel, 1989).

Heidegger's book, *Being and Time*, challenged the Cartesian dualism that separated the subjective from the objective, mind from body and person from world (Guignon, 1983). Heidegger was interested in the ontological question of what it is to be, and he focused on “being‐in‐the world” (Guignon, 1983). Heidegger uses the term *Dasein* to describe the human way of being‐in‐the world. Dasein is a German word that can be defined as existence (Guignon, 1983). Dasein is the human being's ordinary, pre‐theoretical understanding of being, which is reflected in the everyday activities of relating to others and skilful coping (Dreyfus, 1991; Guignon, 1983; Moran, 2000).

For Heidegger, people are thrown into a shared world as members of a particular culture, community and family. Those shared understandings and practices situate them in the world in specific ways (Dreyfus, 1991). For instance, it is expected that parents’ experiences of raising pre‐teen children are implicit in their everyday parenting practices and that these experiences shape their day‐to‐day appraisals of stress and situate them in the world (Benner, 1994; Packer & Addison, 1989). Their parenting practices can only be articulated by considering their past background experiences as members of specific cultures, communities and families. For researchers to understand parents and discover meaning, they must understand how parents of pre‐teen children are constituted by their world and family relationships (Benner, 1994).

According to Heidegger, there are three modes of “being‐in‐the world”: ready‐to‐hand, unready‐to‐hand and present‐at‐hand (Dreyfus, 1991). In the ready‐to‐hand mode, everyday skills and practices are familiar to people as they are actively absorbed in everyday activities. As people engage in activities with expertise, the tools they use become transparent and their bodies act skilfully without conscious thought (Guignon, 1983; Packer & Addison, 1989). This skilful coping is taken for granted because it is pre‐reflexive (Leonard, 1989; Magee, 1987). For instance, the bodily experience of driving a car passes largely unnoticed for a driver with experience. This is because the driver does not have to purposefully think about all of the bodily actions (e.g. steering the wheel or braking) as long as everything is running smoothly (Benner & Wrubel, 1989). The same is thought to be true when parents and pre‐teen children remain connected and have a healthy relationship; everyday activities run smoothly and interactions become transparent (Benner, 1994; Guignon, 1983; Packer & Addison, 1989).

The unready‐to‐hand mode of engagement occurs when a dilemma or breakdown interrupts practical, pre‐reflexive, smoothly running everyday activities. When this occurs, the surrounding conditions that constitute the world come explicitly into view and people become aware of the breakdown (Guignon, 1983; Packer & Addison, 1989). A good way to describe this mode of being is switching from driving an automatic car to a stick shift. An experienced driver of an automatic car might have been driving for many years, so their actions are essentially taken for granted and are often outside the realm of conscious thought. However, suddenly the experienced driver now needs to reflect on all the skills needed to drive the car, such as applying pressure to the gas pedal, pressing down the clutch, and changing gears and then releasing the clutch to reengage the drive. Suddenly, driving becomes a very “cognitive” task, and they start to consider when and how to adjust their driving speed, change gears and break. Basically, driving an automatic car was so automatic that it was often barely noticed; driving only appeared in a conscious way in learning to use a stick shift (Benner & Wrubel, 1989). Similarly, if a parent loses a job, becomes depressed and is unable to fully participate in everyday activities with their pre‐teen child, parent–child interactions may be interrupted and may become problematic (Benner, 1994).

Present‐at‐hand is when a person detaches themselves from the situation to find a solution. Present‐at‐hand is also any experience in which skilful coping is no longer possible, and it forces people to switch to deliberate attention. Implicit action becomes explicit. A person becomes like a scientist observing an experiment and disengages from the dilemma to find a solution. For instance, present‐at‐hand is the way a scientist would examine the characteristics and properties of a rock, measuring its mass and size. It is the mode of staring at something without engagement (Guignon, 1983; Packer & Addison, 1989). Scientists also approach parenting in the present‐at‐hand mode by identifying and measuring variables and their interactions separate from parents’ interpretations and contexts.

Seeking to understand everyday practices and modes of being helps to disclose the shared world and makes it possible to understand what Heidegger calls "the clearing" (Guignon, 1983; Moran & Mooney, 2002). World‐disclosing, or the clearing, is an interpretation or understanding made possible only through a shared background understanding (Guignon, 1983; Moran & Mooney, 2002). Parents learn to skilfully cope with the many stressors and challenges they face as parents based on the background meanings, or practical understandings, of being‐in‐the world as, for instance, residents of a rural community.

### 2.3 Stress and coping

The Lazarus Stress and Coping Paradigm is the framework for designing this semi‐structured interview guide on stress and coping related to parenting pre‐teen children. The Lazarus Stress and Coping Paradigm views stress as residing not in the person or the event but in their interaction (Lazarus & Launier, 1978). Stress can be defined as a person's grasp of the meaning of circumstances for the self when that meaning overloads or surpasses normal adaptive resources (Benner & Wrubel, 1989). The response to stress interrupts practical, smoothly running everyday activities. Coping can be defined as what a person does in response to the disruption (Benner & Wrubel, 1989). This paradigm permits meanings to be identified without transforming them into discrete variables that would destroy the meaning of the situation. This paradigm is complementary to a hermeneutic phenomenological research approach because “stress” and “coping” refer to the dynamic relationship between the person and the world.

Caring for pre‐teen children sets up what counts as stressful for parents and what coping options are available for parents. Parenting stress can be defined as difficulties with the concerns surrounding the parenting role, which were meaning‐dependent and context‐dependent (Lazarus & Folkman, 1984; Benner & Wrubel, 1989). Coping is integral to the parenting experience and the understanding of the parents’ stress, and consequently may transform the original understanding of situations and their concerns. Parental coping can be defined as what is effective for parents to do and is understood as what parents actually do in the situation, including new skills and meanings that parents learned from different situations (Lazarus & Folkman, 1984; Benner & Wrubel, 1989).

## 3 CONSTRUCTING QUESTIONS

Since the Age of Reason, during the 17th and 18th centuries, stories have been dismissed as unscientific and have been discounted as a methodological tool because formal reasoning has been elevated as valid knowledge at the cost of practical rationality (Mishler, 1986). Yet stories continue to be an essential foundation for understanding. For instance, reading a story opens up the world of the narrator, full of the possibilities, concerns, intentions, contradictions, options and impossibilities given in the world of that person. The first‐person account of a story offers an inside‐out viewpoint vital to grasping the terms in which people perceive their life and revealing people's self‐understandings, as well the background conditions that situate activities and contextualize the person (Benner & Wrubel, 1989; Mishler, 1986). Stories from parents of pre‐teen children can recover what formal theories necessarily overlook; namely, how parents of pre‐teen children are social and historical beings.

What distinguishes a hermeneutic phenomenological semi‐structured interview guide from alternatives such as quantitative surveys is the researcher's approach to interviewing diverges in style and form from quantitative interviewing techniques. For example, quantitative surveys are generally carried in a consistent manner to allow the comparison of cases according to a normative structure. The detached style of a quantitative survey and the power of the quantitative researcher to exclusively define legitimate questions and responses that are characteristic of a quantitative survey generally have the practical effect of discouraging contextualized, narrative accounts or stories (Benner & Wrubel, 1989; Mishler, 1986). Standardized quantitative surveys with predetermined and extremely focused lines of questioning limit the essential dialogue, restricting the work of understanding that is the purpose of hermeneutic phenomenological research.

The hermeneutic phenomenological research stance assumes that the study participants’ background meanings provide the basis for understanding. For instance, a parent of a pre‐teen child may take up meanings that are embedded in particular skills and practices of parenting without ever being aware of those meanings. If a parent loses a job, becomes depressed and is unable to fully participate in everyday activities with their pre‐teen child, parent–child interactions may be interrupted and may become problematic (Benner, 1994). Meanings such as these are inherent in parenting practices; they are best studied by examining actual events through stories (SmithBattle, 2014).

Hermeneutic phenomenological research offers a view of the person that is profoundly different from more traditional quantitative notions that are inherently Cartesian. It offers emerging scholars the opportunity to understand the meaningfully rich and complex lived world of those parents of pre‐teen children for whom they care (Benner & Wrubel, 1989; Mishler, 1986). Semi‐structured interview guide questions seek to elicit stories from, for instance, parents of pre‐teen children that reveal the context within which parents and pre‐teen children act, demonstrating how meanings are lived out on the background of shared understandings that develop within a socio‐cultural tradition.

Creating a semi‐structured interview guide for a study employing hermeneutic phenomenological research is an iterative process (SmithBattle, 2014). In a hermeneutic phenomenological research study, the semi‐structured interview guide should encapsulate the aims of what the researcher is trying to reveal, or the ontological, epistemological and methodological stance of the researcher's study (Benner & Wrubel, 1989). “Research questions embed the values, world view, and direction of an inquiry. They also are influential in determining what type of knowledge is going to be generated” (Trede & Higgs, 2009, p. 18). For instance, Phinny (2000) used hermeneutic phenomenological research to examine how people with dementia understand their illness and how their lives changed as a result of dementia.

### 3.1 Stage 1: Identifying the research aims

For much of modern history, medical professionals’ mechanical lens for seeing parents has been used to develop theories on parenting stress and objective resources for coping, using a deductive thought process. Theorists on parenting often treat the parent and child in a machine‐like fashion, and reduce them down to their interactions. This focus on the mechanical constructs and interactions of parents and pre‐teen children, such as parental involvement, engagement, accessibility and responsibility, without understanding the concerns of parents as shaped by context, limits understanding of the world of pre‐teen parents. For example, parenthood not only generates stressors that derive directly from parenting and the parent/pre‐teen child relationship, but it can also exacerbate problems or produce new stressors, such as stress from long‐term illness, occupational stress and financial stress, which may result in parental stress that can have effects on a parent's ability to care for their pre‐teen children.

Experiential meanings that constituted parents’ understanding of raising pre‐teen children (eight to 12 years old) in rural communities were examined using hermeneutic phenomenology research. The researcher examined parenting practices during this developmental stage, as well as the adaptive demands of parenting and the ways parents cope with those demands. Two of the research aims for this study related to stress and coping were to:

- Uncover the challenges of parenting pre‐teen children in rural communities.
- Describe the resources that support parents in raising pre‐teen children in rural communities.

The overall goal in developing this semi‐structured interview guide on stress and coping related to parenting pre‐teen children was to discover how personal background meanings and interpersonal concerns shape parents’ day‐to‐day stress appraisals and coping with parenting pre‐teen children. Additionally, the researcher decided the sample for this study would consist of a convenience sample of 16 married or cohabitating parenting dyads from a rural Midwest community with at least one pre‐teen child, born any time from 2008–2011. The researcher wanted this semi‐structured interview on stress and coping related to parenting pre‐teen children to last approximately 60 min.

### 3.2 Stage 2: Literature review

**Figure 1:**
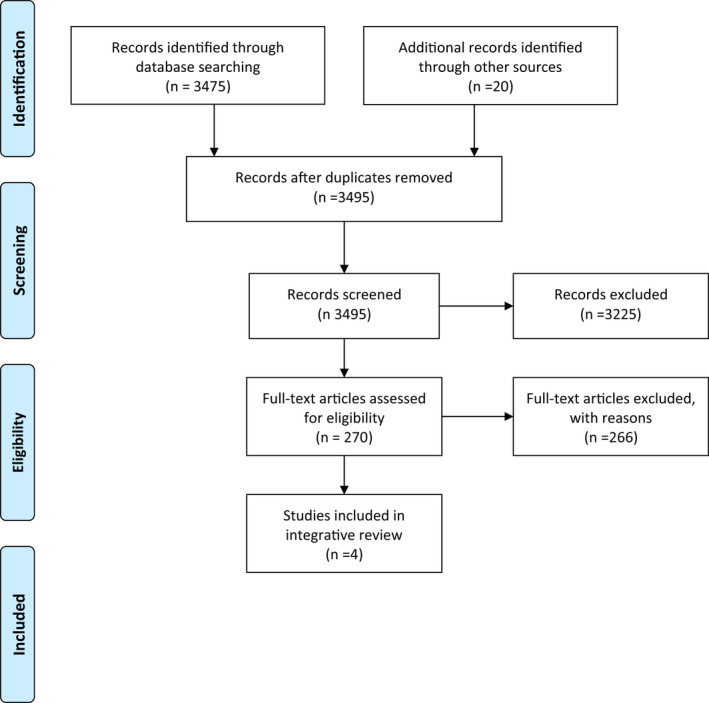
PRISMA flow diagram. *Note*. Search terms: “hermeneutic*” or “phenomenology*” and “stress*” or “coping*” and “interview*” Search period: January 1970 through September of 2019. PRISMA = Preferred Reporting Items of Systematic Reviews and Meta‐Analyses (Liberati et al., 2009)

After deciding on the purpose of the study and research goal(s), and determining which study participants would provide the best information to answer the research question based on inclusion and exclusion criteria, relevant literature related to stress and coping was reviewed. A medical librarian‐assisted literature search was performed through the databases Ovid MEDLINE, CIHAHL, ERIC, SCOPUS, Web of Science, JSTOR, Education Source, PsyINFO and ProQuest. The search terms included the words “hermeneutic*” or “phenomenology*” and “stress*” or “coping*” and “interview*” in the title, abstract or keywords. The literature search was conducted in 2019 and examined articles from 1 January 1970 to 30 June 2019. The search yielded 3,475 articles after duplicates were deleted. An ancestry search of the reference list of manuscripts and authors was also completed; 20 additional manuscripts were found. Inclusion criteria were studies using hermeneutic phenomenological research to evaluate stress and coping. Manuscripts were excluded if they did not include a semi‐structured interview guide (Figure 1).

Unfortunately, it is uncommon for researchers to publish how they created interview guides, and it can be difficult for some emerging scholars to find good examples of semi‐structured interview guides for a hermeneutic phenomenological research study. However, some dissertations provide examples of interview guides.

Many researchers’ dissertations have constructed interview guides to investigate stress and coping. For instance, SmithBattle (1992) examined the teenager's transition to mothering as shaped by the family's caregiving practices and the mother's participation in a defining community. She examined personal and family understandings related to caring for a young mother and her child as they are expressed in actual caregiving practices and rituals, and in stress and coping incidents (SmithBattle, 1992). Pohlman (2003) used semi‐structured interview guides to examine the meanings, concerns and practices of fathers of pre‐term infants. Stressful aspects of their experience were located outside the NICU and involved the juggling act between work, hospital visits and home; paradoxically, work was also noted as a coping resource for fathers (Pohlman, 2003). Another researcher sought to identify patterns and indicators of pre‐clinical disability among older women. A hermeneutic phenomenological research approach was taken to explore embodiment, taken‐for‐granted bodily sensations and coping practices (Lorenz, 2007). Finally, Fyle‐Thorpe (2015) examined the experiences of low‐income, non‐resident African American fathers with regard to parenting and depression. She revealed the challenges and barriers to parenting among low‐income non‐resident African American fathers (Fyle‐Thorpe, 2015).

A table was constructed with types of guiding questions, including (a) warm up questions; (b) core questions; (c) probing questions; and (d) wrap up questions related to stress and coping (Table 1). Warm up questions do not have to directly relate to the aims of the researcher's study (although they might), but help with rapport‐building, which will put the researcher and study participant more at ease with one another, allowing the rest of the interview to progress smoothly. Core questions are more difficult or potentially embarrassing questions. The goal is to tap into study participants’ experiences and expertise. Probing questions elicit more detailed and elaborate responses to key questions. For a study employing hermeneutic phenomenological research, the more details the study participants share, the better. Finally, wrap up questions provide closure for an interview and prevent the interview from ending abruptly.

**Table 1:** Guiding questions

Type of question	Definition	Purpose	Example of stress and coping questions from the literature
Warm up questions	Warm up questions are the first questions of an interview that may or may not be related to the content of the overall research questions.	These questions are used to help initiate the interview and help participants start talking about their experiences.	In the past few months, your sister/daughter had a baby. What has it been like to be a grandparent/aunt or uncle? (SmithBattle, 1992) Describe any health problems you now have or have had in the last year? (Lorenz, 2007) Have there been any big changes in your lives since my last visit a couple weeks ago? (SmithBattle, 1992)
Core questions	Core questions directly relate to the information the researcher wants to know.	These questions are used to help participants talk openly and more specifically about the topic.	I am interested in learning about what it is like to become a father of a pre‐term infant and what aspects of having a pre‐term infant are difficult. Can you tell me about a recent event that stands out for you as being particularly difficult? (Pohlman, 2003) I would like you to describe a situation that stands out for your family as being particularly difficult or stressful in living with or caring for a young mother and her baby. (SmithBattle, 1992) Can you tell me what helps you cope with discrimination or obstacles with being African American? (Who in your family or friends helps you cope?) (Fyle‐Thorpe, 2015). How does your child cope with difficult situations? (Fyle‐Thorpe, 2015). What keeps you going when you have these kinds of difficulties? (Fyle‐Thorpe, 2015).
Probing questions	Probing questions are questions that ask for more details about a particular aspect of the core questions	These questions are used to answer particular aspects of the core interview questions and obtain greater detail about responses from the participants.	Tell me what happened. (Pohlman, 2003) What led up to the situation? (Pohlman, 2003) What were your thoughts, feelings, and reactions to the situation? What were your priorities during the incident? (Pohlman, 2003) What did you do? (Pohlman, 2003) How did you feel afterwards? (Pohlman, 2003) What else did you consider doing? (Pohlman, 2003) Who was most helpful to you in this situation? (Pohlman, 2003) Looking back on it now, is there anything you would do differently? (Pohlman, 2003) What did you learn about yourself in this situation? What did you learn about your baby? (Pohlman, 2003) What was most helpful to you in this situation? (Lorenz, 2007)
Wrap up questions	Wrap up questions are the last questions of an interview.	These questions are used to provide closure to an interview and prevent the interview from ending abruptly.	Is there anything else you want to add that my questions have not addressed? (Fyle‐Thorpe, 2015). Is there any circumstance you want to discuss that we have not talked about? (Fyle‐Thorpe, 2015)

The researcher made sure all questions put in the table were open ended, neutral, and clear, and avoided leading language. In addition, the researcher only included example questions that used familiar language and avoided jargon. Table 1 gives details of the types of guiding questions including warm up questions, core questions, probing questions and wrap up questions (SmithBattle, 2014).

### 3.3 Stage 3: Writing the questions

**Figure 2:**
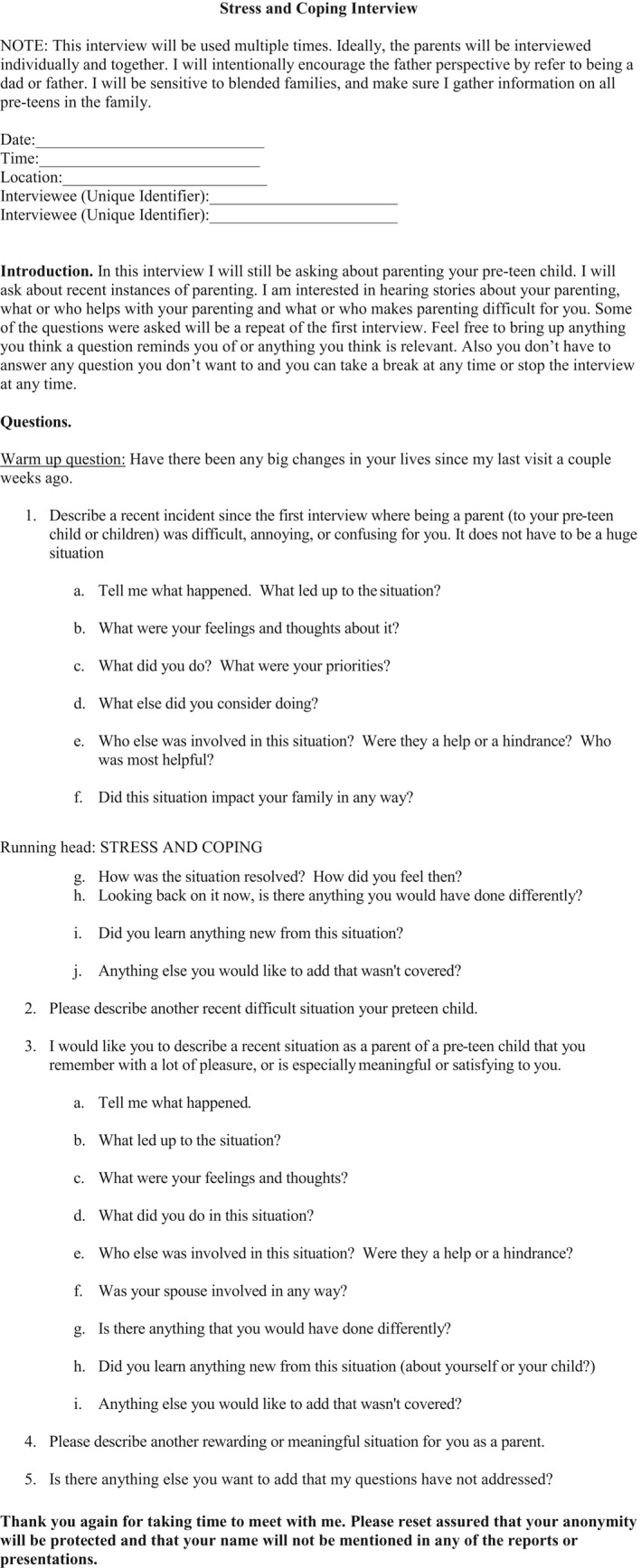
Stress and coping interview

The questions for this stress and coping interview guide were based on the work of other hermeneutic phenomenological researchers (Fyle‐Thorpe, 2015; Lorenz, 2007; Pohlman, 2005; SmithBattle, 1992). An initial list of questions was developed based on Table 1. First, warm up questions were developed (Figure 2). These questions were used to help parents feel more at ease talking about potentially stressful situations.

Second, core questions were developed. These questions were adapted to fit the study participants, parents of pre‐teen children living in rural communities. When using a hermeneutic phenomenological research approach for an interview guide about stress and coping, it is important to remember the primary source of knowledge is everyday practical activity. The study participant's perspective is paramount and core questions need to encourage detailed stories of lived experiences. In contrast to a quantitative survey, the core questions for this semi‐structured interview guide on stress and coping seek to elicit stories about specific incidents, events and situations directed at human behaviour which became the text analogue that was studied and interpreted in order to discover hidden or obscured meaning. It is also important for emerging scholars to remember that the core questions are not fixed, standardized interviews that guarantee minimal responses amenable to discrete coding, interview protocols but core questions serve as flexible guides that encourage a dialogue that provides rich, thick descriptions.

Finally, probing questions were developed to elicit more detailed stories that would provide specific examples of how personal background meanings and concerns shape parents’ day‐to‐day stress appraisals and coping with parenting pre‐teen children in rural communities. It is important for emerging scholars to remember that meaning is often hidden because it is so pervasive and so taken for granted that it goes unnoticed. Since everyday lived experiences are so taken for granted as to go unnoticed, it is often through breakdowns that the researcher achieves flashes of insight into the lived world. Therefore, probes can assist in eliciting rich, descriptive information about the research setting, study participants, and their stories of their experiences. The probing questions were developed more as suggestions that may help the study participant to elaborate a detailed story of what they actually did, thought and felt about specific situations as they evolved and with enough detail so that the context of the situation would be fully described. Emerging scholars need to remember that the relevance of the probing questions may change from study participant to study participant, and some probing questions may not be asked.

After the initial list of questions was developed, the list was reviewed for language and sequencing. This semi‐structured interview guide on stress and coping begins with a warm up question. The researcher took care to make sure this was something the study participant could answer easily but that wouldn't take too long. Next, the researcher considered the flow of the interview to make sure it was logical. What issues should be asked about first? What questions should come next, and would seem more or less “natural”? The most challenging or possibly upsetting core questions were asked towards the end of the interview, after rapport had been built. The last question, or the wrap up question, was intentionally chosen to leave the study participant feeling encouraged, heard, or otherwise glad they participated in the interview (SmithBattle, 2014).

### 3.4 Stage 4: Assessing rigour

Assessing the rigour of a hermeneutic phenomenological semi‐structured interview guide requires different criteria than those for assessing the validity and reliability of a quantitative survey (Creswell, 2016; Streubert & Carpenter, 2011). The rigour of a hermeneutic phenomenological semi‐structured interview guide must be consistent with understanding human experience (Benner et al., 1996). The researcher promoted the rigour of this semi‐structured interview guide in several different ways. First, the researcher participated in interpretive reading groups as a way to refine, challenge and validate initial semi‐structured interview guide questions (Benner, 1994). Colleagues read and analysed the questions. These sessions provided insight and feedback that assisted in refining and confirming the questions (Benner, 1994; Morse, 2015).

Then, the semi‐structured interview guide was used in mock interviews and feedback was given. A mock interview is an experience that includes both professors and doctoral students who practise together and help each other refine skills for interviewing (SmithBattle, 2014). Conducting mock interviews allowed the researcher to make sure questions were simple and that they were not asking more than one question at a time (SmithBattle, 2014). Conducting mock interviews allowed the researcher to begin to understand which questions prompted the longest answers from the study participants. The researcher found some of the questions could only be answered with a few words; these questions were removed. The researcher crafted the questions so that study participants would be encouraged to answer as authentically and completely as possible. During the mock interviews, the researcher thought through alternative ways of answering a few of the questions, such as through observation. Limitations and biases of the semi‐structured interview guide questions were also discussed with the researcher's advisor. It took several mock interviews to judge the correct length of the semi‐structured interview guide.

To ensure the rigour of this semi‐structured interview guide, the researcher also considered sensitivity to context, transparency and generalizability. Sensitivity to context was established through demonstrating sensitivity to the existing literature and the socio‐cultural setting of the study when writing the interview questions (Rodgers & Cowles, 1993). Transparency refers to how clearly the stages of the research process are described in the write‐up (Rodgers & Cowles, 1993). The semi‐structured interview guide was created using a table, which established an audit trail as a way to enhance transparency. The principle of generalizability or transferability is controversial in hermeneutic phenomenological research because the goal is to provide a rich, contextualized understanding of the human experience. The principle of generalizability in hermeneutic phenomenological research reflects how well or sensitively a piece of research is conducted, and whether or not it tells the reader something clinically useful (Creswell, 2016; Morse, 2015; Rodgers & Cowles, 1993). To achieve generalizability, the researcher sought to create rich, high‐quality questions that would elicit descriptive information about the research setting, study participants and their experiences. This will assist future researchers in making appropriate judgements about the proximal similarity of study contexts and their own environments.

### 3.5 Limitations

When hermeneutic phenomenological researchers create a semi‐structured interview guide and then conduct interviews and observations, they are thrown forward from their forestructure of understanding into the experiences of their study participants (Packer & Addison, 1989). This process is called the hermeneutic circle. The hermeneutic circle consists of forestructure and biases (pre‐suppositions), which are positive and negative (Packer & Addison, 1989). As the researcher moves in this circle of understanding, the researcher begins with their biases or what they already know (from personal experience, theory, research findings) and returns as they gain an appreciation of where they began (forestructure) and where their initial understanding may confirm or diverge from their study participants’ understanding and experience (Gadamer, 1975). While their initial biases helped them to enter the field, study participants’ data may enlarge their understanding and eventually, the hermeneutic phenomenological researcher experiences a fusion of horizons with study participants as the researcher better understands how one's own forestructure has shaped the original aims, research questions and early interpretation of the data (Gadamer, 1975). Therefore, limitations and biases of the semi‐structured interview guide questions were discussed with the researcher's advisor as the semi‐structured interview guide was developed. The researcher's own forestructure was also acknowledged as the semi‐structured interview guide was developed. Acknowledging forestructure is consistent with the hermeneutic phenomenological research process (Crist & Tanner, 2003; Finlay, 2006; Morse, 2015).

## 4 DISCUSSION

The goal of this semi‐structured interview guide on stress and coping related to parenting pre‐teen children was to encourage study participants to tell stories of their experiences so that meaning in context could be captured from personal accounts of the everyday world (Mishler, 1979). The semi‐structured interview guide was used to initiate conversation and encourage dialogue with parents of pre‐teen children. None of the questions in this semi‐structured interview guide abstracted experience and life events from their context of the parents’ stories. Parents were asked to describe a recent difficult or challenging situation, followed by a recent meaningful episode. Careful probing questions assisted parents in elaborating their thoughts, feelings and actions in self‐selected stress and coping situations, as they occurred in context. The probing, clarifying questions changed during conversations with parents, and the interviewer sought to help them provide detailed stories of what they did, thought and felt about specific situations as they occurred. The researcher sought to elicit as much detail about their stories as possible so that the researcher could more fully understand the parents’ thoughts and actions about particular situations (Benner & Wrubel, 1989; Mishler, 1986). The researcher followed the parents’ lead in the conversations because what parents of pre‐teen children chose to talk about reflected their practical understanding of their world of parenting pre‐teen children. Language, as noted by Guignon (1983), who interpreted Heidegger's philosophy, constitutes both the understanding and situatedness of our everyday being and lays out the possibilities of grasping the world.

Hermeneutic phenomenological research analysis began after the first interview and continued as data were collected. Interpretation of transcripts from interviews did not proceed in a linear or step‐like predetermined manner but was a circular process that unfolded as the researcher's provisional understanding of the transcripts from the study participants’ stories grew in depth from multiple readings and analysis (Benner & Wrubel, 1989). Initially, the transcripts were read by the researcher to obtain an overall understanding of the study participants’ meanings of parenting; then, texts were compared and contrasted to identify differences and similarities in meanings and practices (Allen et al., 1986). Through systematic analysis of the whole, the researcher gained new perspectives and depths of understanding. The researcher used this insight to examine parts of the whole and then reconsider the whole in light of the understanding gained from the parts. Each transcript was read multiple times to obtain a sense of each parent's situation and to gain an overall sense of their meanings (Sandelowski, 1995; Streubert & Carpenter, 2011). This phase raised numerous questions as narrative descriptions of stress and coping activities related to parenting pre‐teen children provided contrasts and similarities between whole cases and between differing perspectives of the mothers and fathers. This process followed this part‐whole strategy until the researcher was satisfied with the depth of understanding. Recurring themes relating to stress and coping were identified in this first phase and modified with successive readings.

Emerging scholars need to be aware that research questions may be transformed during the process of conducting interviews, as practices and meaning emerge from the perspectives of various study participants. Emerging scholars should anticipate that the semi‐structured interview guide will be revised as the study continues, as new questions emerge from interviews (SmithBattle, 2014). For instance, the original aims of one researcher's study were to: “(a) increase understanding of what the experience of receiving family care means to elders; (b) show how the personal, social, and cultural meanings of health and illness of elders shape their interpretation of receiving family care; and (c) identify patterns about receiving family care that are shared across gender, age, and culture” (Crist & Tanner, 2003). After it was obvious that having family care did not mean the experience was a defining aspect of the lives of the first study participants engaging in the interviews, the aim was modified (Crist & Tanner, 2003).

## 5 CONCLUSION

Semi‐structured interviewing can be an impactful tool for emerging scholars to use to understand how personal background meanings and interpersonal concerns shape background meanings and concerns related to stress and coping. To clarify this approach to creating a stress and coping interview guide for emerging scholars, recommendations for the essential steps to follow in order to best create semi‐structured interviews related to stress and coping were suggested. Emerging scholars should expect that their ability to develop semi‐structured interview guides will be improved and refined as they continue to conduct studies that advance the science and practice of nursing.

## CONFLICT OF INTEREST

No conflict of interest has been declared by the author.

## ETHICAL APPROVAL

This semi‐structured interview guide received Institutional Review Board (IRB) approval prior to interviews being conducted. Study participants had an opportunity to ask questions or raise concerns with the researcher prior to starting the interview. During this time, the researcher reiterated that study participants had the option to refuse to answer any question and could withdraw from the study at any time. Additionally, study participants were notified that, with their consent, audio recordings and notes were collected throughout the interview.

## Data Availability

The authors confirm that the data supporting the findings of this study are available within the article [and/or] its supplementary materials.

## References

[nop2778-bib-0001] Benner, P. (1994). The tradition and skill of interpretive phenomenology in studying health, illness, and caring practices. In P. Benner (Ed.), Interpretive phenomenology: Embodiment, caring, and ethics in health and illness (pp. 99–127). Sage.

[nop2778-bib-0002] Benner, P. A., Tanner, C. A., & Chesla, C. A. (1996). Expertise in nursing practice: Caring, clinical judgment, and ethics (pp. 351–372). Springer.

[nop2778-bib-0003] Benner, P., & Wrubel, J. (1989). The primacy of caring: Stress and coping in health and illness, 1st ed. Addison‐Wesley.

[nop2778-bib-0004] Berman, M. (1981). The birth of modern scientific consciousness. The Reenchantment of the World (pp. 27–46). Cornell University Press.

[nop2778-bib-0005] Creswell, J. W. (2016). 30 essential skills for the qualitative researcher. Sage.

[nop2778-bib-0006] Crist, J. D., & Tanner, C. A. (2003). Interpretation/analysis methods in hermeneutic interpretive phenomenology. Nursing Research, 52(3), 202–205. 10.1097/00006199-200305000-00011 12792262

[nop2778-bib-0007] Dreyfus, H. L. (1991). Being‐in‐the‐World: A commentary on Heidegger’s Being and Time. Division I. : The MIT Press.

[nop2778-bib-0503] Finlay, L. (2006). ‘Rigour’, ‘ethical integrity’ or ‘artistry’? Reflexively reviewing criteria for evaluating qualitative research. British Journal of Occupational Therapy, 69, 319–326.

[nop2778-bib-0008] Fyle‐Thorpe, O. (2015). The experiences of low income non‐resident African American fathers with parenting and depressive symptoms. Retrieved from http://search.proquest.com/docview/288332793?accountid=8065Randolph

[nop2778-bib-0009] Gadamer, H.‐G. (1975). Truth and method. The Seabury Press.

[nop2778-bib-0010] Guignon, C. B. (1983). Heidegger and the problem of knowledge. Hackett Publishing.

[nop2778-bib-0011] Lazarus, R. S., & Launier, R. (1978). Stress‐related transactions between person and environment. In L. A. Pervin, & M. Lewis (Eds.), Perspectives in interactional psychology. Plenum Press.

[nop2778-bib-0501] Lazarus, R., & Folkman, S. (1984). Stress. Appraisal and coping.New York: Springer.

[nop2778-bib-0012] Leder, D. (1984). Medicine and paradigms of embodiment. Journal of Medicine and Philosophy, 9, 29–43. 10.1093/jmp/9.1.29. 6726100

[nop2778-bib-0013] Leder, D. (1992). A tale of two bodies: The Cartesian corpse and the lived body. In D. Leder (Ed.), The body in medical thought and practice (pp. 17–36). Kluwer Academic Publishers.

[nop2778-bib-0014] Leonard, V. W. (1989). A Heideggerian phenomenologic perspective on the concept of the person. Advances in Nursing Science, 11(4), 40–55. 10.1097/00012272-198907000-00008. 2500894

[nop2778-bib-0015] Liberati, A., Altman, D. G., Tetzlaff, J., Mulrow, C., Gøtzsche, P. C., Ioannidis, J. P. A., Clarke, M., Devereaux, P. J., Kleijnen, J., & Moher, D. (2009). The PRISMA statement for reporting systematic reviews and meta‐analyses of studies that evaluate health care interventions: Explanation and elaboration. PLoS Medicine, 6(7), e1000100. 10.1371/journal.pmed.1000100. 19621070 PMC2707010

[nop2778-bib-0016] Lorenz, R. (2007). Women’s perspectives on aging: Coping with change. Retrieved from http://search.proquest.com/docview/288332793?accountid=8065Randolph

[nop2778-bib-0017] Magee, B. (1987). Husserl, Heidegger and modern existentialism: Dialogue with Hubert Dreyfus. In: The great philosophers: An introduction to Western philosophy (pp. 254–277). Oxford University Press.

[nop2778-bib-0018] McConnell‐Henry, T., Chapman, Y., & Francis, K. (2009). Husserl and Heidegger: Exploring the disparity. International Journal of Nursing Practice, 15, 7–15. 10.1111/j.1440-172X.2008.01724.x. 19187164

[nop2778-bib-0019] Mishler, E. (1986). Research interviewing: Context and narrative. Harvard University Press.

[nop2778-bib-0504] Mishler, E. G. (1979). Meaning in context: Is there any other kind? Harvard Education Review, 49, 1–19.

[nop2778-bib-0020] Moran, D. (2000). Introduction to Phenomenology. Routledge.

[nop2778-bib-0021] Moran, D., & Mooney, T. (Eds.) (2002). The phenomenology reader (pp. 288–307). Routledge.

[nop2778-bib-0022] Morse, J. (2015). Critical analysis of strategies for determining rigor in qualitative inquiry. Qualitative Health Research, 25, 1212–1222. 10.1177/1049732315588501 26184336

[nop2778-bib-0023] Packer, M. J., & Addison, R. B. (1989). Introduction. In M. J. Packer, & R. B. Addison (Eds.), Entering the circle: Hermeneutic investigation in psychology (pp. 13–36). Statue University of New York.

[nop2778-bib-0024] Phinny, J. (2000). The persistence of meaning in the midst of breakdown: An interpretive phenomenological account of symptom experience in dementia. Retrieved from http://search.proquest.com/docview/288332793?accountid=8065Randolph

[nop2778-bib-0025] Pohlman, S. (2003). When worlds collide: The meanings of work and fathering among fathers of premature infants. Retrieved from http://search.proquest.com/docview/288332793?accountid=8065Randolph

[nop2778-bib-0502] Pohlman, S. (2005). The primacy of work and fathering preterm infants: findings from an interpretive phenomenological study. Advances in Neonatal Care: Official Journal of the National Association of Neonatal Nurses, 5(4), 204–216. 10.1016/j.adnc.2005.03.002 16084478

[nop2778-bib-0027] Rodgers, B. L., & Cowles, K. V. (1993). The qualitative research audit trail: A complex collection of documentation. Research in Nursing and Health, 16, 219–226. 10.1002/nur.4770160309 8497674

[nop2778-bib-0028] Sandelowski, M. (1995). Focus on qualitative methods qualitative analysis: What it is and how to begin. Research in Nursing & Health, 18, 371–375. 10.1002/nur.4770180411. 7624531

[nop2778-bib-0029] SmithBattle, L. (1992). Caring for teenage mothers and their children: Narratives of self and ethics of intergenerational caregiving. Retrieved from http://search.proquest.com/docview/288332793?accountid=8065Randolph

[nop2778-bib-0030] Smithbattle, L. (2014). Developing students’ qualitative muscles in an introductory methods course. International Journal of Nursing Education Scholarship, 11(1), 129–136. 10.1515/ijnes-2014-0025 25178908

[nop2778-bib-0031] SmithBattle, L. (2018). Housing trajectories of teen mothers and their families over 28 years. American Journal of Orthopsychiatry, 89(2), 258–267. 10.1037/ort0000347 30010361

[nop2778-bib-0032] Streubert, H. J., & Carpenter, D. R. (2011). Qualitative research in nursing, 5th ed. Lippincott.

[nop2778-bib-0033] Trede, F., & Higgs, J. (2009). Framing research questions and writing philosophically: The role of framing research questions. In J. Higgs, D. Horsfall, & S. Grace (Eds.), Writing qualitative research question on practice (pp. 13–25). Sense.

[nop2778-bib-0034] Wrubel, J., Benner, P., & Lazarus, R. (1981). Social competence from the perspective of stress and coping. In: J. D. Wine, & M. D. Smye (Eds.). Social Competence (pp. 61–99). Guilford Press.

